# Supplementary figures and images for: Membrane contact probability: An essential and predictive character for the structural and functional studies of membrane proteins
Source: PLoS Comput Biol. 2022 Mar 30;18(3):e1009972. doi: 10.1371/journal.pcbi.1009972 (PMC9000120; doi:10.1371/journal.pcbi.1009972)

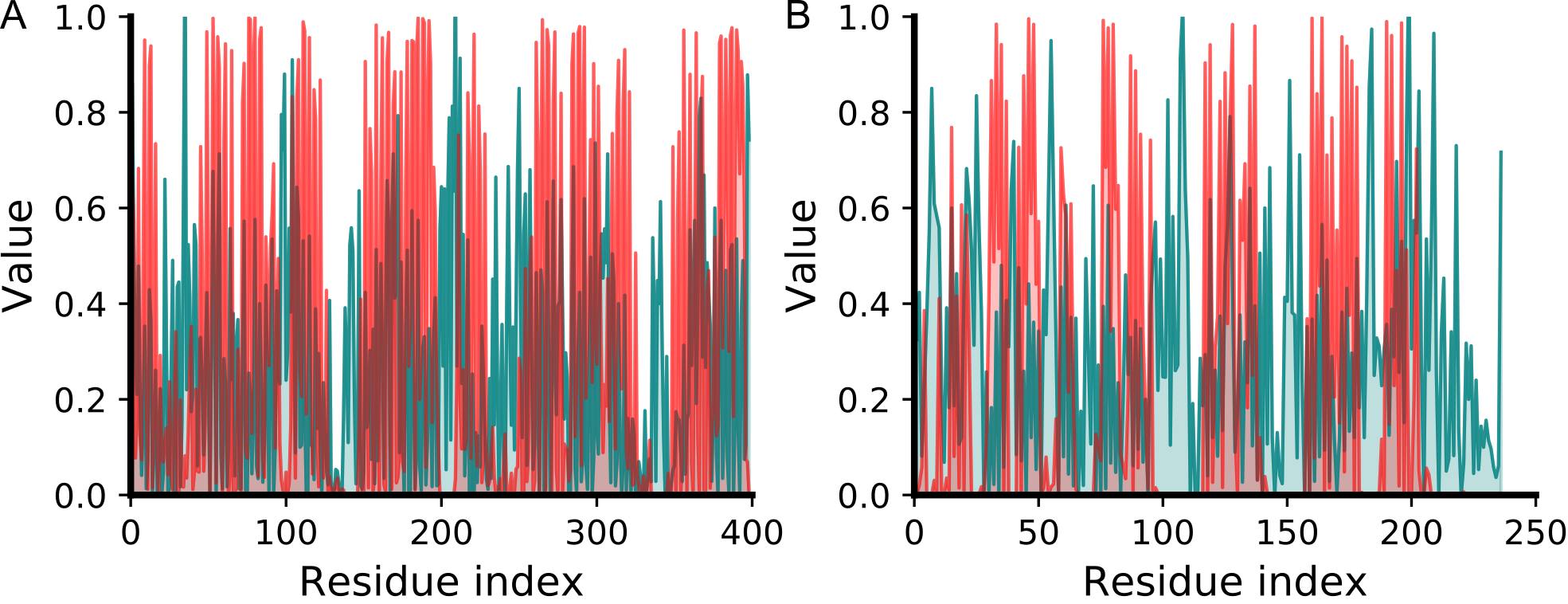

Supplement: S1 Fig — The MCP and outer exposure results are shown as red and teal lines, respectively. According to DSSP, the buried residues are defined to have an RSA value of 0-0.1, so the outer surface residues have a RSA values of 0.1-1. We calculated the percentage of membrane-contacting residues with high MCP values (>0.5) lying in the outer residues with RSA >0.1. The value is 84.0% for 5aym, and 88.9% for 4e1t. Therefore, most of the membrane-contacting residues predicted by MCP are outer-surface residues. (TIF) [file pcbi.1009972.s001.tif]

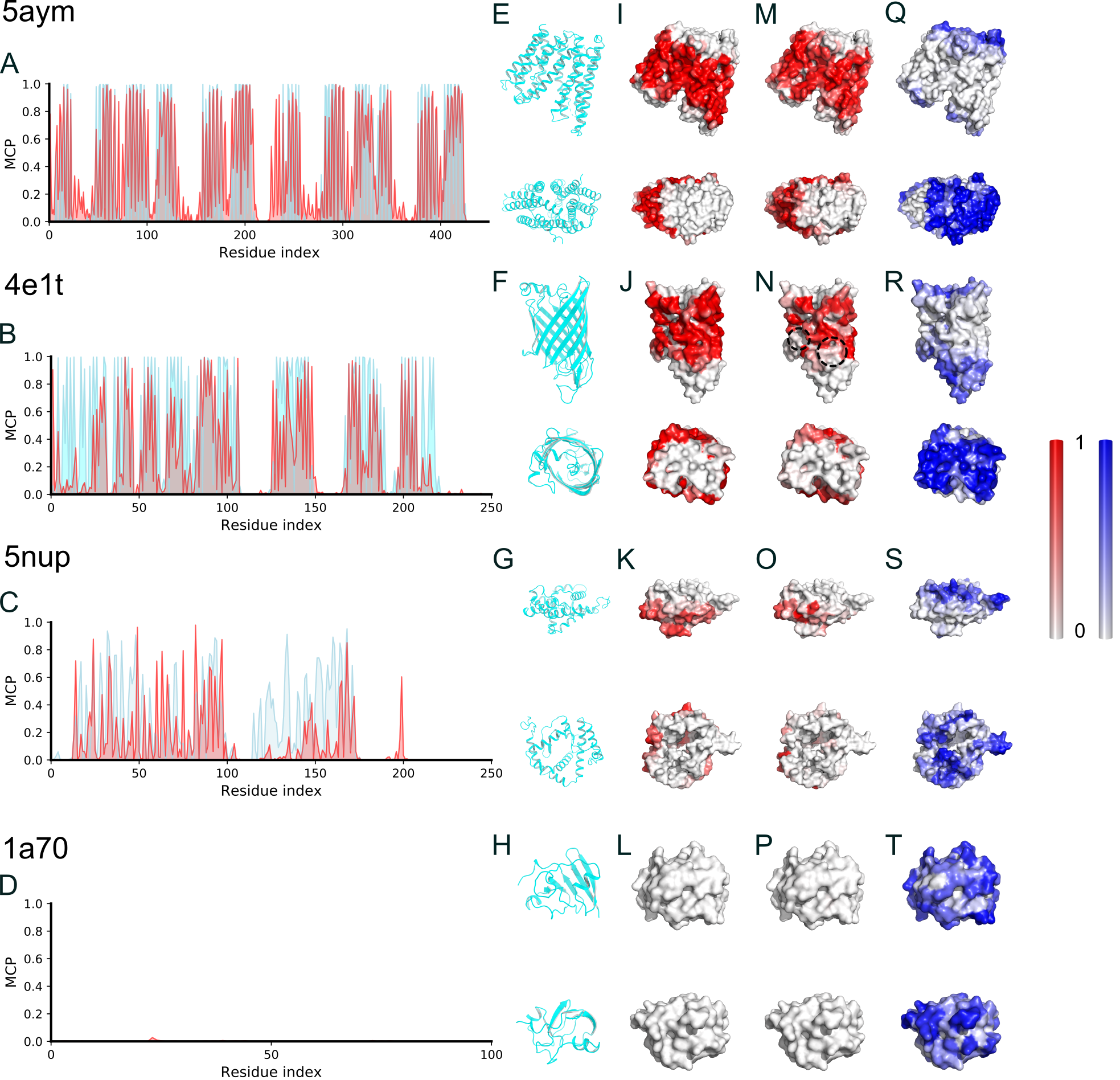

Supplement: S2 Fig — (A-D) Comparison between the observation (cyan) and the prediction (red) of the MCPs. (E-H), Side and top views of the four representative proteins. (I-L), The outer surface of the representative proteins, colored according to the observed MCP values obtained from MD simulations. (M-P), Similar to (I-L), but colored according to the predicted MCP values. (Q-T), Similar to (I-L), but colored according to the predicted SA values by RaptorX. (TIF) [file pcbi.1009972.s002.tif]

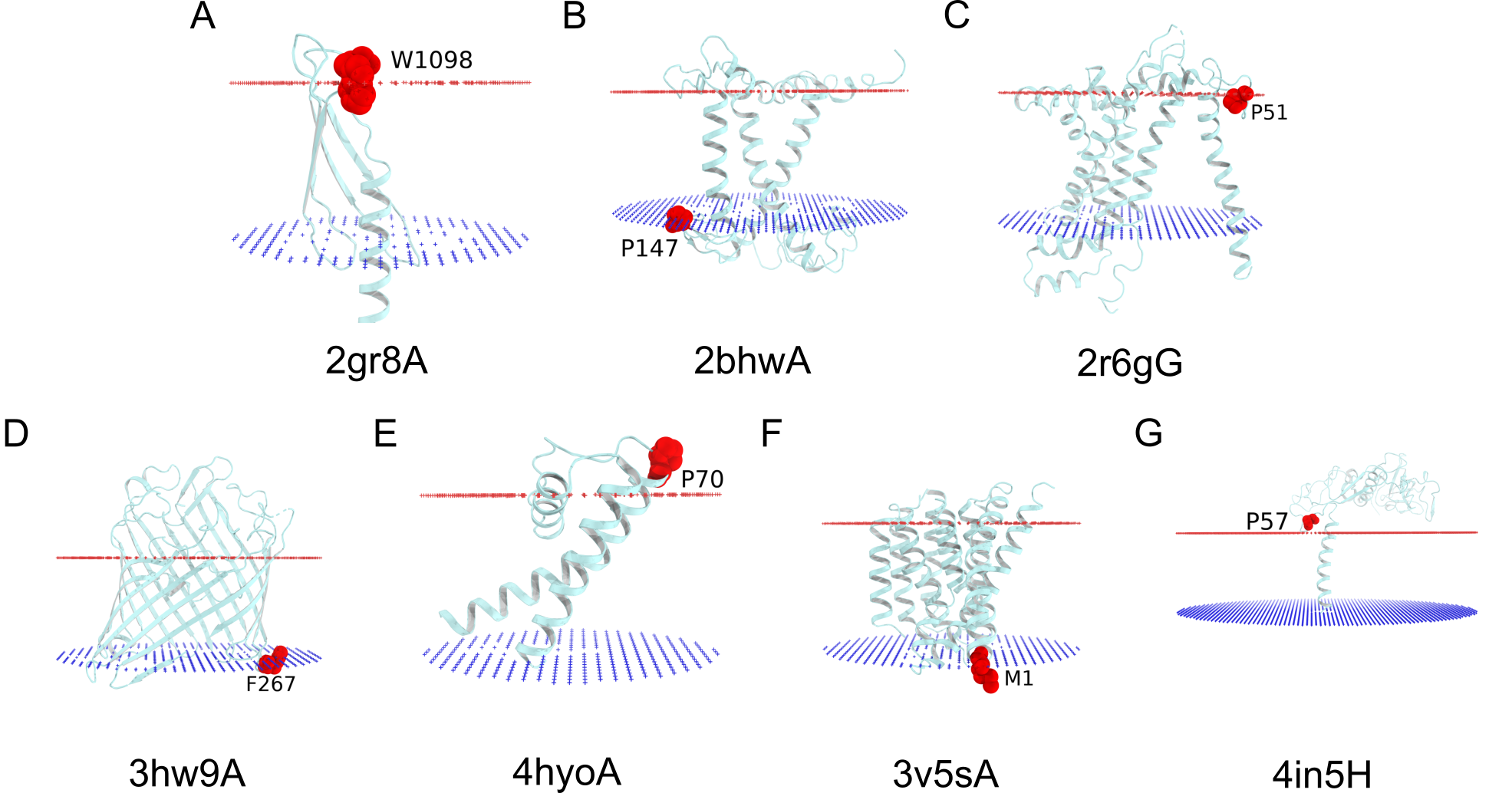

Supplement: S3 Fig — The hydrophobic boundaries of the lipid bilayer are represented by the red and blue pseudo-atoms, indicating the outer and inner surfaces of the bilayer, respectively. (TIF) [file pcbi.1009972.s003.tif]

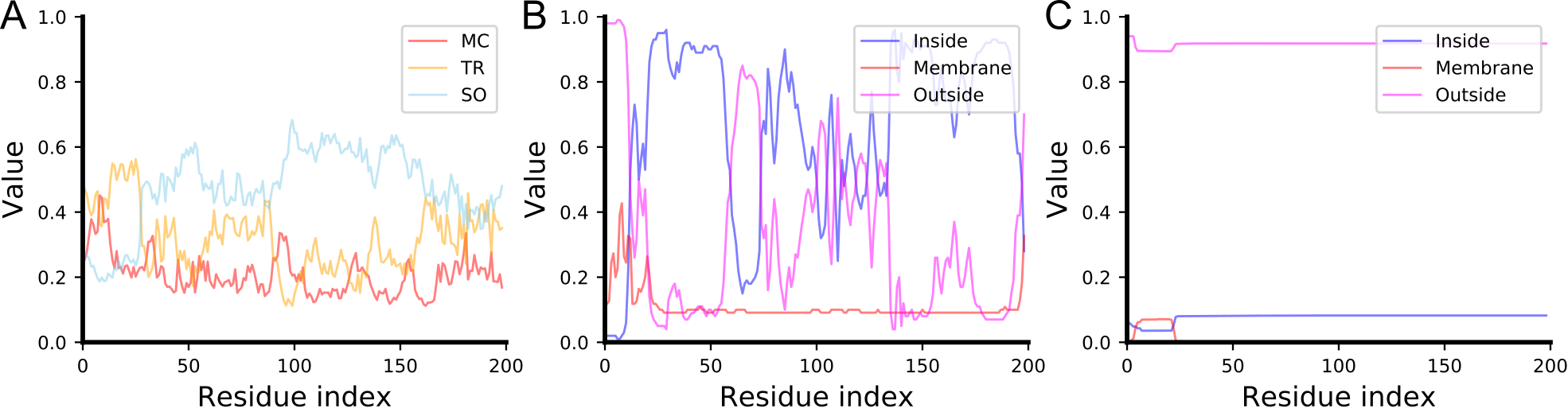

Supplement: S4 Fig — (A) The results of BCL::Jufo9D (red for membrane core (MC), sky blue for transition region (TR), and orange for solution (SO)). (B) The results of OCTOPUS (red for membrane, blue for inside, and fuchsia for outside). (C) The results of TMHMM (red for membrane, blue for inside, and fuchsia for outside). (TIF) [file pcbi.1009972.s004.tif]

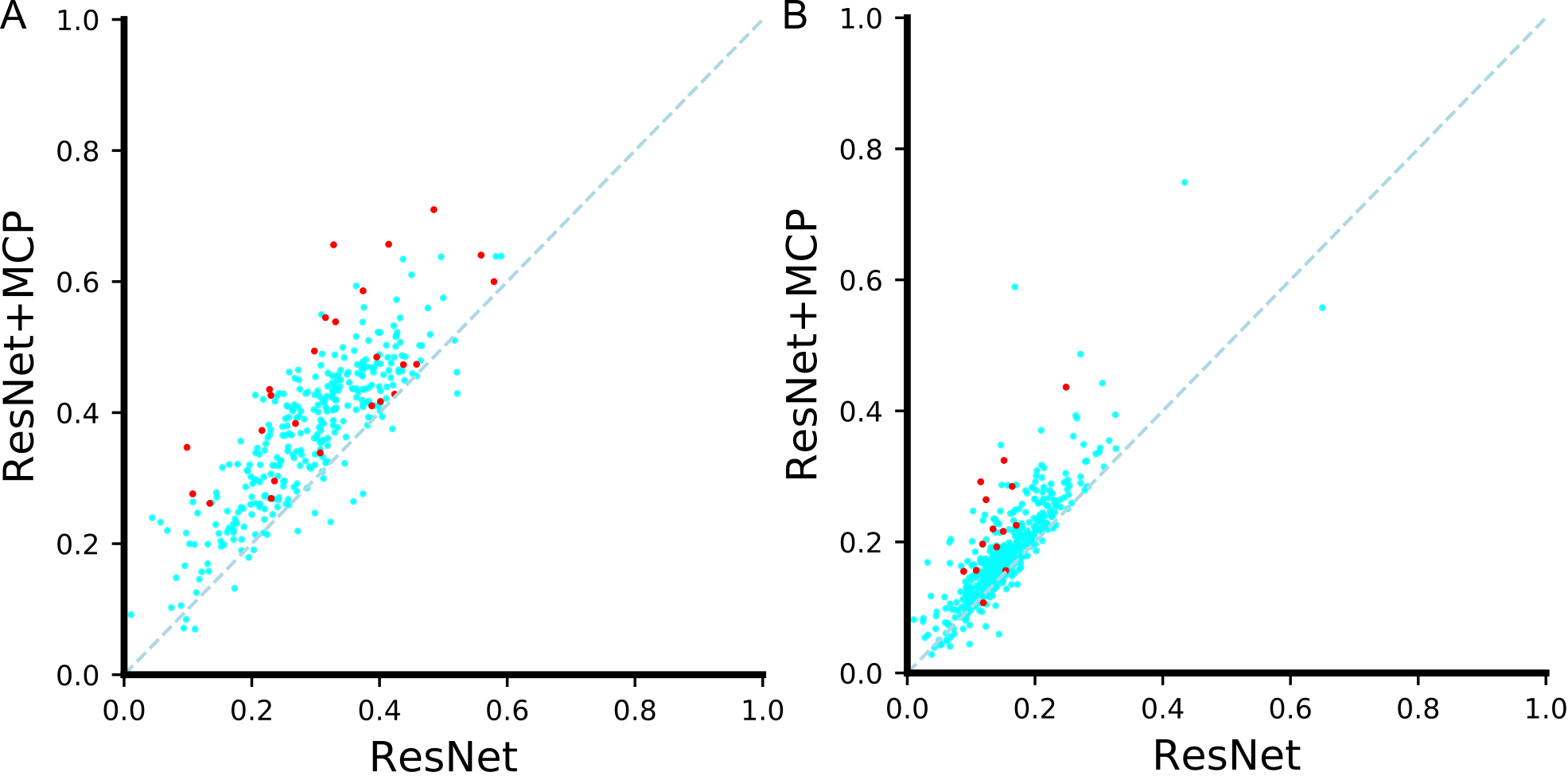

Supplement: S5 Fig — The original ResNet predictor (X-axis) vs our MCP-incorporated ResNet predictor (Y-axis) for the 327-protein dataset (A) and 495-protein dataset (B), respectively. The dashed line is the function y = x. Each point represents a test protein, with red points for membrane proteins. (TIF) [file pcbi.1009972.s005.tif]

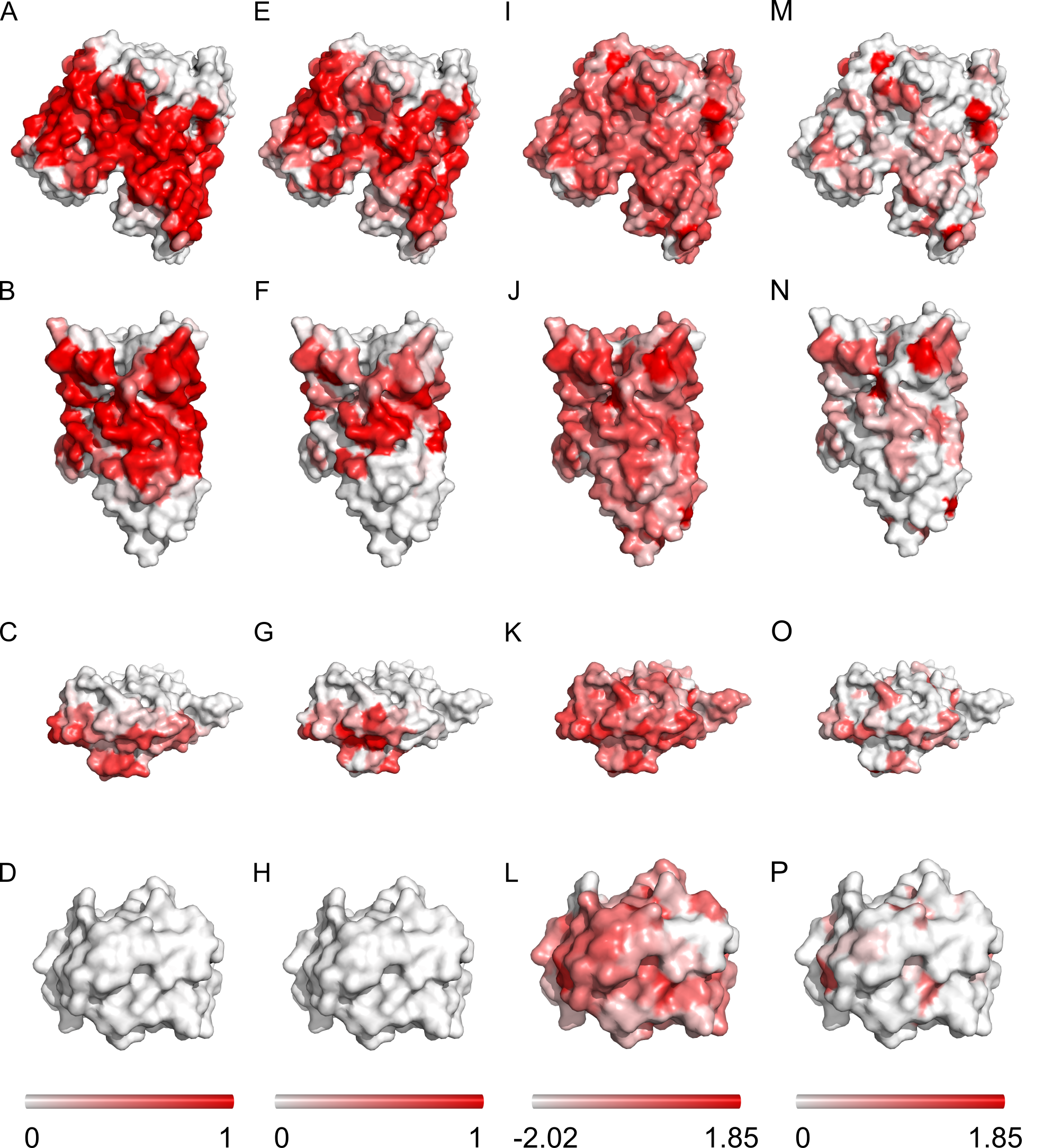

Supplement: S6 Fig — (A-D), The colored outer surfaces of the four representative proteins presented in Figs 2 and 4, according to the observed MCP values from MD simulations. (E-H), Similar to (A-D), but colored according to the predicted MCP values. (I-L), Similar to (A-D), but colored according to the value of the Wimley-White hydrophobicity scales. (M-P), Similar to (I-L), but with a different color bar. (TIF) [file pcbi.1009972.s006.tif]

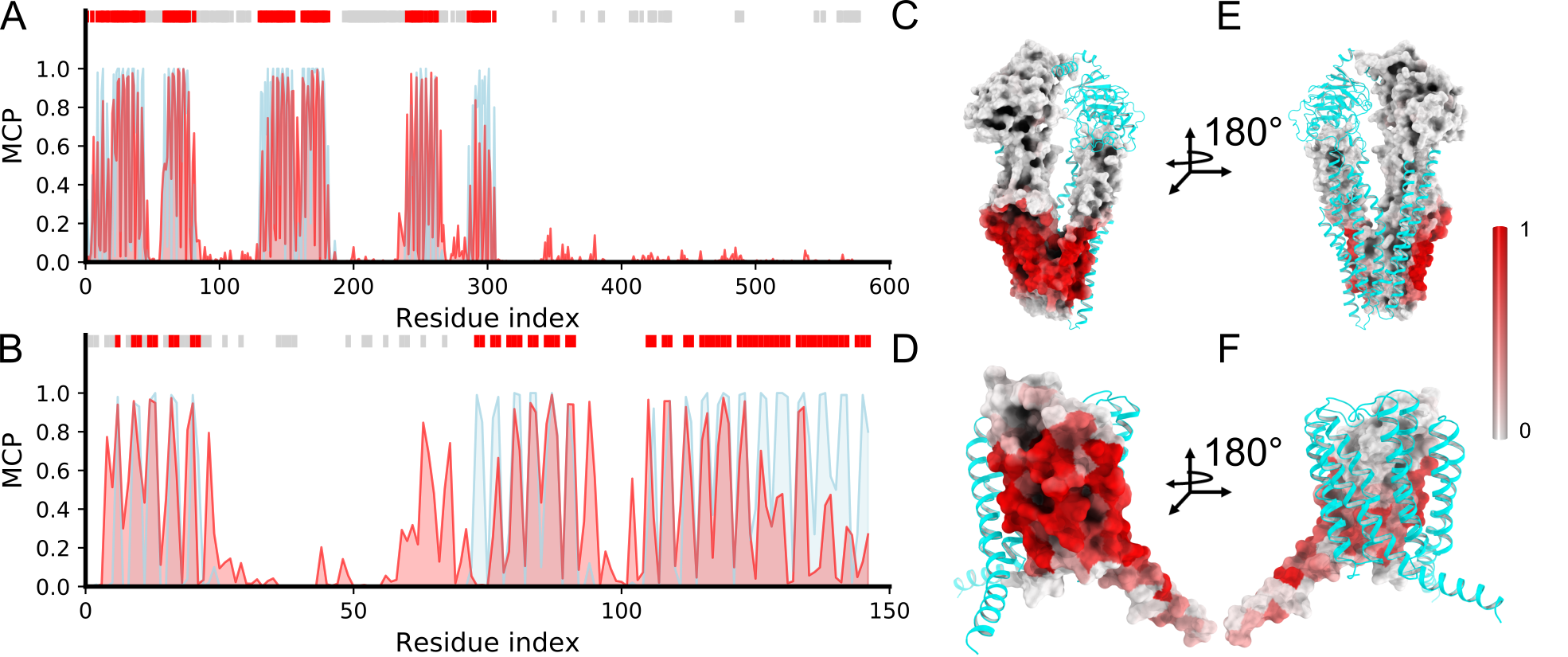

Supplement: S7 Fig — The two complex proteins were in the test dataset with the overall prediction PCCs of 0.84 and 0.61, representing one of the good and one of the poor predictions, respectively. (A-B) Comparison between the observation (light blue) and the prediction (red) of MCP. The horizontal bars on the top of the panels indicate the regions of the protein-membrane (red) and protein-protein interfaces (gray). (C-D) The outer surface of the two proteins colored according to the predicted MCP values. (E-F) Similar to (C-D), but from another view. As can be seen, the protein-protein interface residues (gray bar) overall show low MCP values than those at the protein-membrane interfaces (red bar). The protein-membrane interface residues were defined by MCP >0.2, while the protein-protein interface residues were defined with the script ‘InterfaceResidues’ of Pymol. (TIF) [file pcbi.1009972.s007.tif]

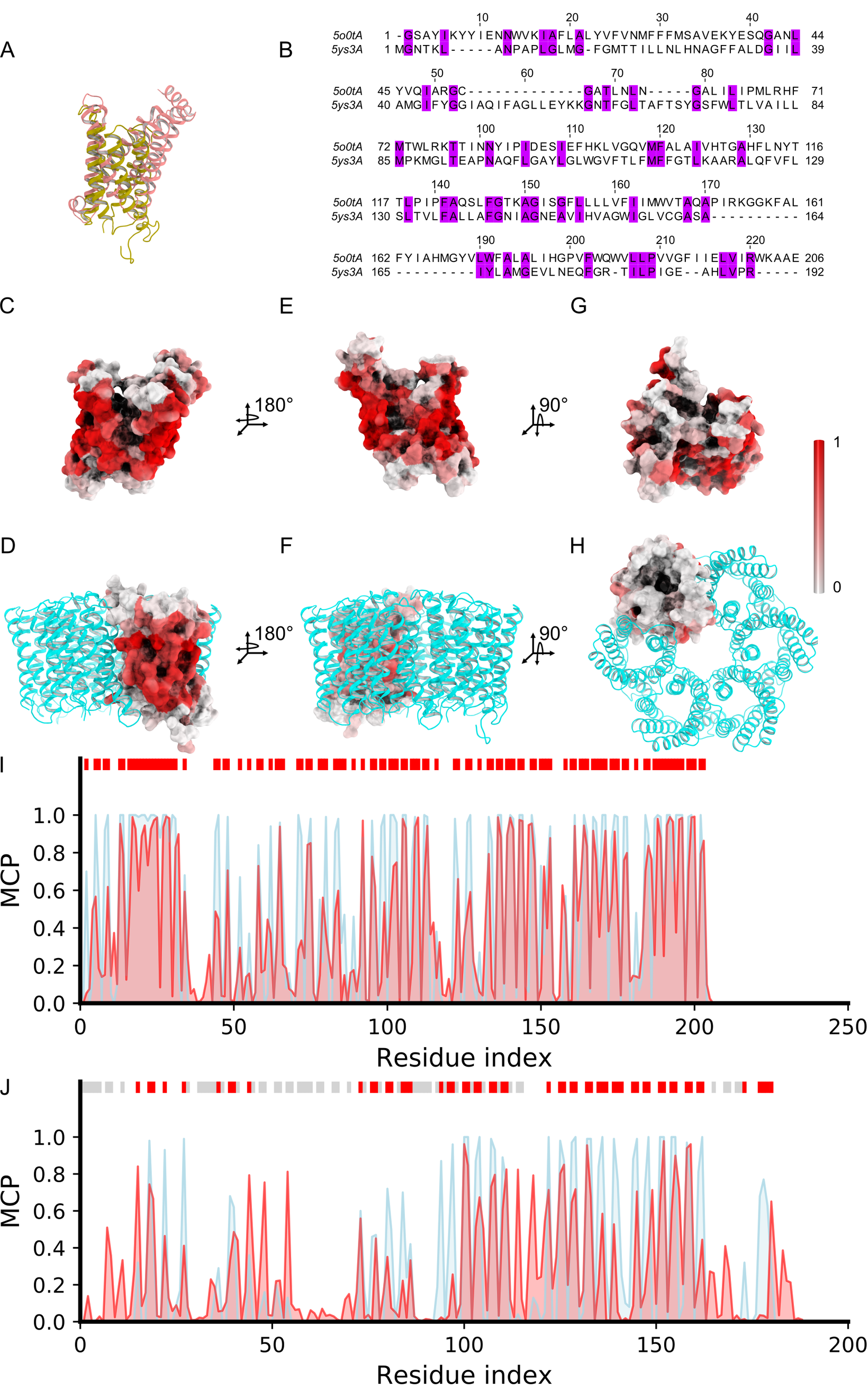

Supplement: S8 Fig — (A) The structure alignment of the two proteins showed a similar fold (TM-score = 0.45, normalized by the length of 5ys3). (B) The sequence alignment of the two proteins (sequence similarity = 20.9%, calculated by MUSCLE). (C-D) The outer surface of the two proteins colored according to the predicted MCP values. (E-F) Similar to (C-D), but from another side view. (G-H) Similar to (C-D), but from the top view.(I-J) Comparison between the MD observation (light blue) and the prediction (red) of MCP. The horizontal bars on the top of the panels indicate the regions of the protein-membrane (red) and protein-protein interfaces (gray). As can be seen, the protein-protein interface residues (gray bar) show overall low MCP values than those at the protein-membrane interfaces (red bar) in the transmembrane region. The protein-membrane interface residues were defined by MCP >0.2, while the protein-protein interface residues were defined with the script ‘InterfaceResidues’ of Pymol. (TIF) [file pcbi.1009972.s008.tif]

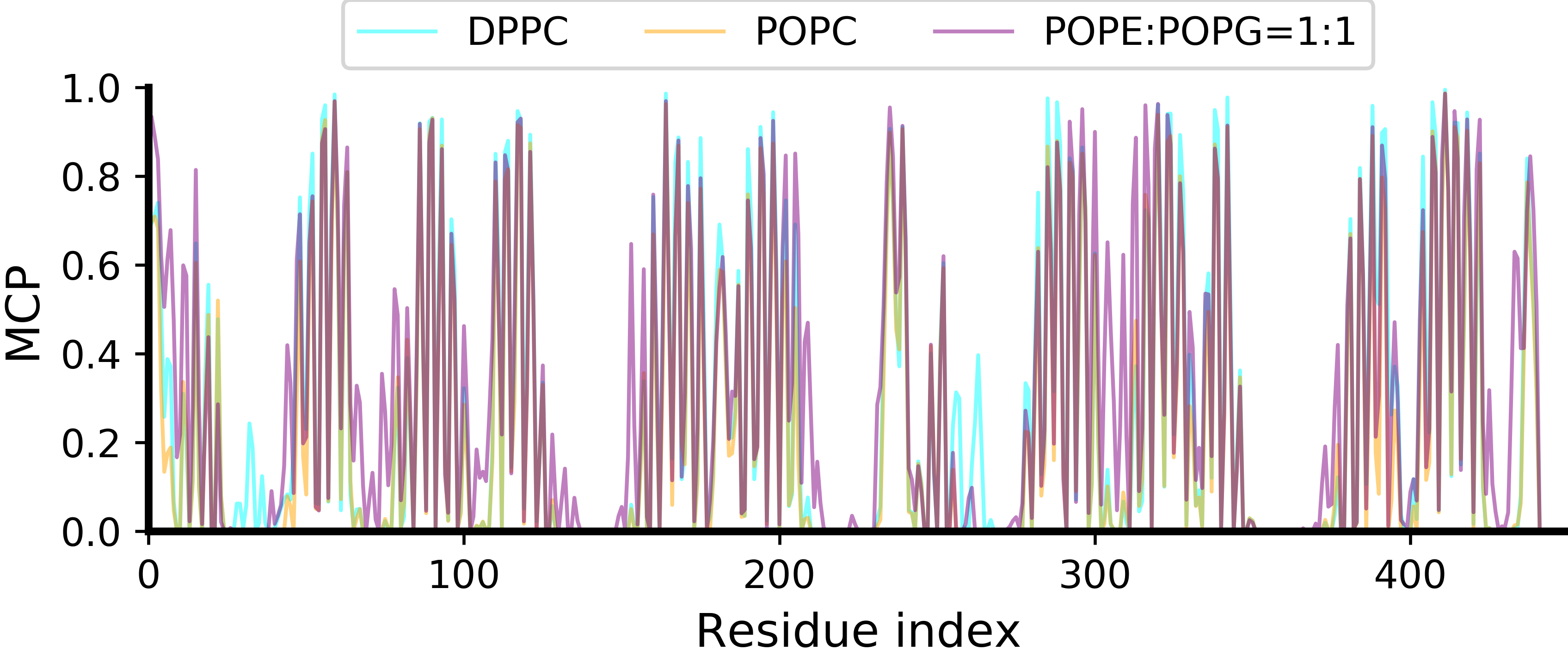

Supplement: S9 Fig — These results show that the saturation, head group and net charge of the lipids have minor impacts on the observed MCP in the hydrophobic core region, and the differences are mostly located at the membrane-water interfaces. (TIF) [file pcbi.1009972.s009.tif]

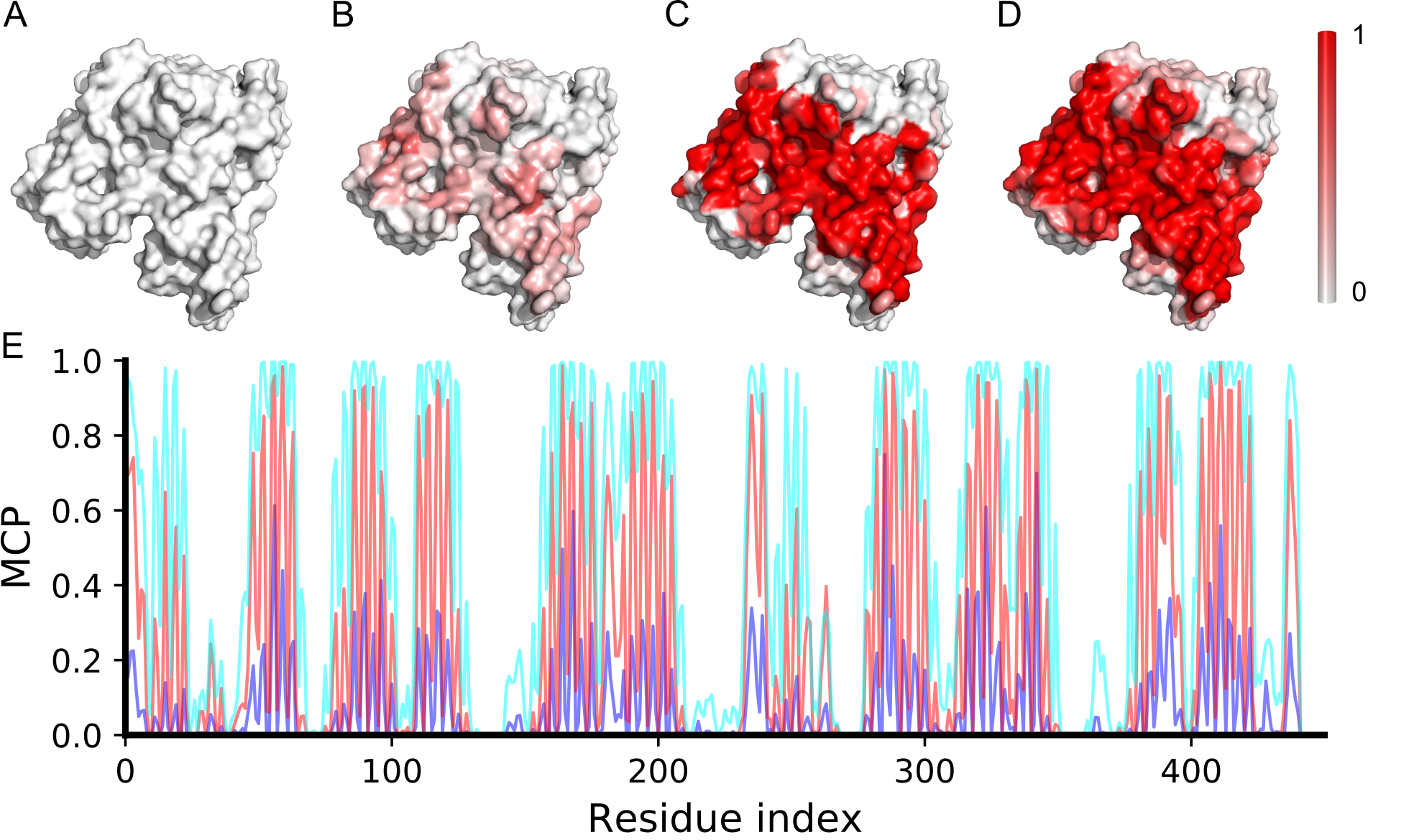

Supplement: S10 Fig — (A-D), The colored outer surface of the protein (PDB ID: 5aym) according to the observed MCP values obtained from MD simulations with different cutoff values of 4, 5, 6, and 8 Å, respectively. As can be seen, a cutoff value smaller than 6 Å would lead to weak signals, while a cutoff value larger than 8 Å would start to overestimate the transmembrane region. (E), Comparison between the observed MCPs with different cutoff values; black, blue, red, and cyan lines for 4, 5, 6, and 8 Å, respectively. (TIF) [file pcbi.1009972.s010.tif]

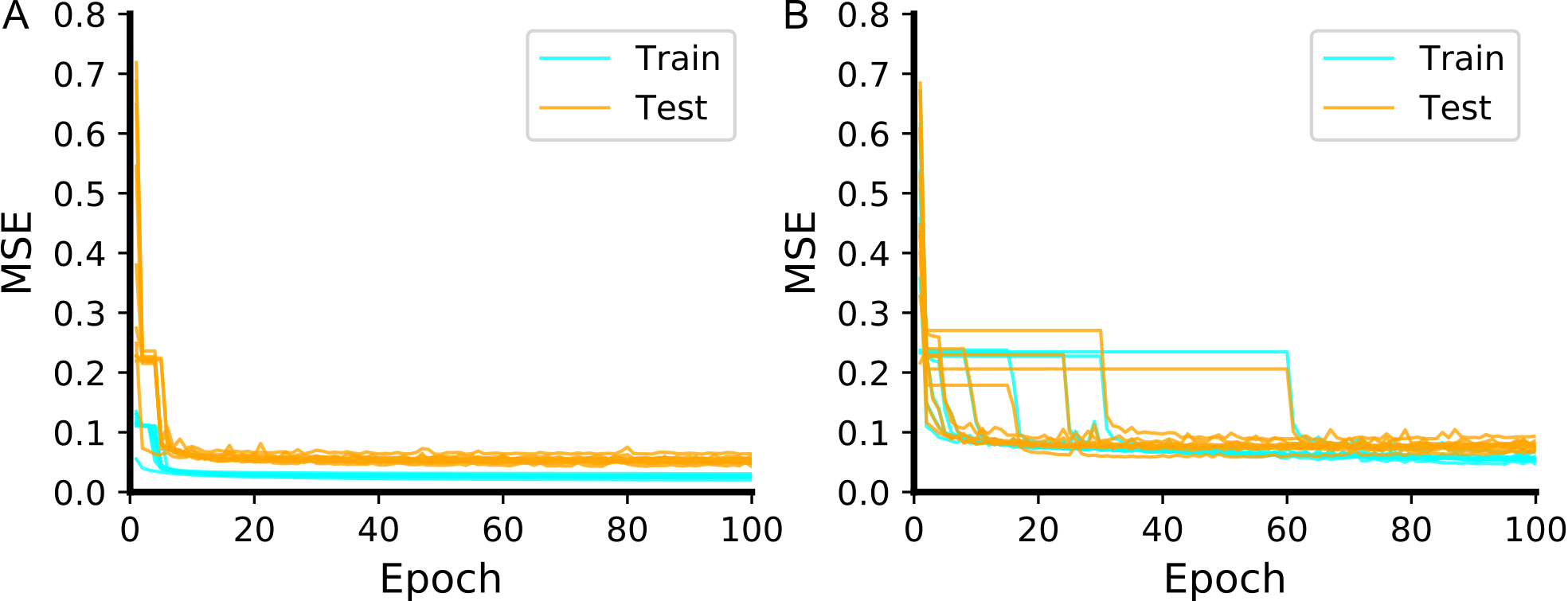

Supplement: S11 Fig — The left panel was obtained with the MCP-Large dataset, and the right panel with the MCP-Small dataset. (TIF) [file pcbi.1009972.s011.tif]

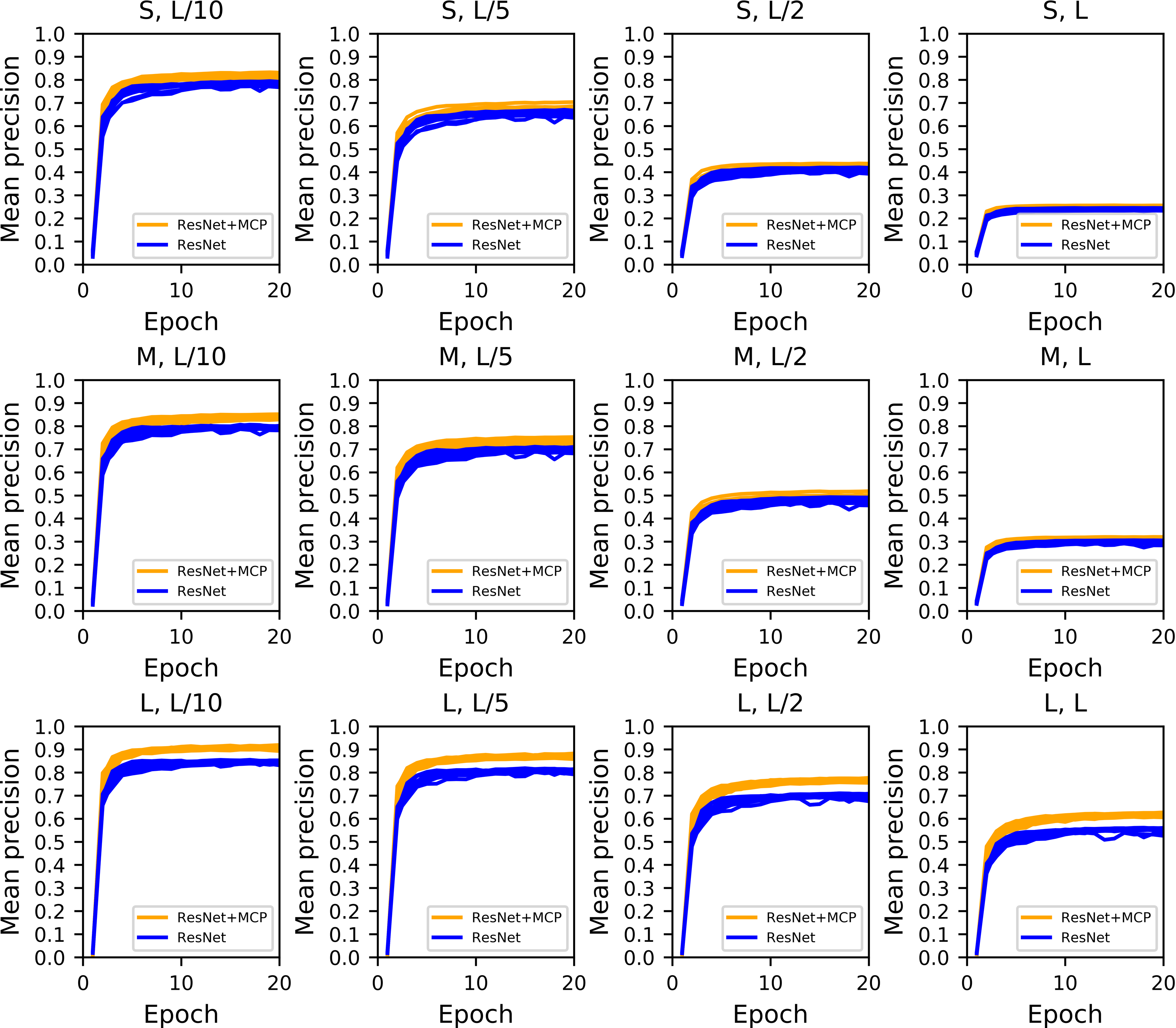

Supplement: S12 Fig — (TIF) [file pcbi.1009972.s012.tif]
