# Supplementary material for: Membrane contact probability: An essential and predictive character for the structural and functional studies of membrane proteins
Source: PLoS Comput Biol. 2022 Mar 30;18(3):e1009972. doi: 10.1371/journal.pcbi.1009972 (PMC9000120; doi:10.1371/journal.pcbi.1009972)
Supplement: S1 Table — (DOCX) [file pcbi.1009972.s014.docx]

**Table S1: The related prediction methods for membrane protein features.**

| Method | Year | Model | Prediction | URL |
| --- | --- | --- | --- | --- |
| $\alpha-$helical transmembrane protein | | | | |
| TMHMM2.0 [1] | 2001 | HMM | Topology | https://services.healthtech.dtu.dk/service.php?TMHMM-2.0 |
| MEMSAT3 [2] | 2007 | NN |  | http://bioinf.cs.ucl.ac.uk/?id=756 |
| OCTOPUS [3] | 2008 | HMM, ANN |  | http://topcons.net/ |
| SCAMPI2 [4] | 2016 | HMM |  | http://scampi.bioinfo.se/ |
| MemBrain3.0 [5] | 2020 | ResNet |  | http://www.csbio.sjtu.edu.cn/bioinf/MemBrain/ |
| PolyPhobius [6] | 2005 | HMM | Topology  Signal peptide | http://phobius.sbc.su.se/poly.html |
| Philius [7] | 2008 | DBN |  | http://www.yeastrc.org/philius/pages/philius/runPhilius.jsp |
| SPOCTOPUS [8] | 2008 | HMM, ANN |  | http://topcons.net/ |
| TOPCONS2 [9] | 2009 | HMM |  | http://topcons.net/ |
| MEMSAT-SVM [10] | 2009 | SVM |  | http://bioinf.cs.ucl.ac.uk/psipred/ |
| CCTOP [11] | 2015 | HMM |  | http://cctop.enzim.ttk.mta.hu/ |
| MPRAP [12] | 2010 | SVM | Surface accessibility | https://mprap.cbr.su.se/ |
| $\beta-$barrel transmembrane protein | | | | |
| PROFtmb [13] | 2006 | HMM | Topology | https://www.predictprotein.org/ |
| BetAware [14] | 2013 | ELM, GRHCRF |  | http://betaware.biocomp.unibo.it/BetAware |
| BOCTOPUS2 [15] | 2016 | SVM, HMM |  | http://boctopus.bioinfo.se/ |
| PRED-TMBB2 [16] | 2016 | HMM |  | http://www.compgen.org/tools/PRED-TMBB2/ |
| $\alpha-$helical transmembrane protein or $\beta-$barrel transmembrane protein | | | | |
| BCL::Jufo9D [17] | 2013 | ANN | Transmembrane spans | http://meilerlab.org/index.php/servers/show?s_id=5 |
| mp_lipid_acc [18] | 2017 | Concave hull algorithm | Lipid accessibility | https://rosie.graylab.jhu.edu/mp_lipid_acc/submit |

HMM: Hidden Markov model; NN: Neural network; DBN: Dynamic Bayesian network; ANN: Artificial neural network; SVM: Support vector machine; ELM: Extreme learning machine; GRHCRF: Grammatical-restrained hidden conditional random field.

**Reference**

1. Krogh A, Larsson B, Heijne GV, Sonnhammer EJJoMB. Krogh A, Larsson B, Von Heijne G, Sonnhammer ELL.. Predicting transmembrane protein topology with a hidden Markov model: application to complete genomes. J Mol Biol 305: 567-580. 2001;305(3):567-80.

2. Jones DT. Improving the accuracy of transmembrane protein topology prediction using evolutionary information. Bioinformatics. 2007;23(5):538-44. doi: 10.1093/bioinformatics/btl677.

3. Håkan V, Arne EJB. OCTOPUS: improving topology prediction by two-track ANN-based preference scores and an extended topological grammar. 2008;24(15):1662-8.

4. Peters C, Tsirigos KD, Shu N, Elofsson A. Improved topology prediction using the terminal hydrophobic helices rule. Bioinformatics. 2016;32(8):1158-62. doi: 10.1093/bioinformatics/btv709.

5. Feng S-H, Zhang W-X, Yang J, Yang Y, Shen H-B. Topology Prediction Improvement of α-helical Transmembrane Proteins Through Helix-tail Modeling and Multiscale Deep Learning Fusion. Journal of Molecular Biology. 2020;432(4):1279-96. doi: <https://doi.org/10.1016/j.jmb.2019.12.007>.

6. Käll L, Krogh A, Sonnhammer ELL. An HMM posterior decoder for sequence feature prediction that includes homology information. Bioinformatics. 2005;21(suppl_1):i251-i7. doi: 10.1093/bioinformatics/bti1014.

7. Reynolds SM, Käll L, Riffle ME, Bilmes JA, Noble WS. Transmembrane Topology and Signal Peptide Prediction Using Dynamic Bayesian Networks. PLOS Computational Biology. 2008;4(11):e1000213. doi: 10.1371/journal.pcbi.1000213.

8. Viklund H, Bernsel A, Skwark M, Elofsson A. SPOCTOPUS: a combined predictor of signal peptides and membrane protein topology. Bioinformatics. 2008;24(24):2928-9. doi: 10.1093/bioinformatics/btn550.

9. Bernsel A, Viklund H, Hennerdal A, Elofsson A. TOPCONS: consensus prediction of membrane protein topology. Nucleic Acids Research. 2009;37(suppl_2):W465-W8. doi: 10.1093/nar/gkp363.

10. Nugent T, Jones DT. Transmembrane protein topology prediction using support vector machines. BMC Bioinformatics. 2009;10(1):159. doi: 10.1186/1471-2105-10-159.

11. Dobson L, Reményi I, Tusnády GE. CCTOP: a Consensus Constrained TOPology prediction web server. Nucleic Acids Research. 2015;43(W1):W408-W12. doi: 10.1093/nar/gkv451.

12. Illergård K, Callegari S, Elofsson A. MPRAP: An accessibility predictor for a-helical transmem-brane proteins that performs well inside and outside the membrane. BMC Bioinformatics. 2010;11(1):333. doi: 10.1186/1471-2105-11-333.

13. Bigelow H, Rost B. PROFtmb: a web server for predicting bacterial transmembrane beta barrel proteins. Nucleic Acids Research. 2006;34(suppl_2):W186-W8. doi: 10.1093/nar/gkl262.

14. Savojardo C, Fariselli P, Casadio R. BETAWARE: a machine-learning tool to detect and predict transmembrane beta-barrel proteins in prokaryotes. Bioinformatics. 2013;29(4):504-5. doi: 10.1093/bioinformatics/bts728.

15. Hayat S, Peters C, Shu N, Tsirigos KD, Elofsson A. Inclusion of dyad-repeat pattern improves topology prediction of transmembrane β-barrel proteins. Bioinformatics. 2016;32(10):1571-3. doi: 10.1093/bioinformatics/btw025.

16. Tsirigos KD, Elofsson A, Bagos PG. PRED-TMBB2: improved topology prediction and detection of beta-barrel outer membrane proteins. Bioinformatics. 2016;32(17):i665-i71. doi: 10.1093/bioinformatics/btw444.

17. Leman JK, Mueller R, Karakas M, Woetzel N, Meiler J. Simultaneous prediction of protein secondary structure and transmembrane spans. Proteins. 2013;81(7):1127-40. Epub 2013/04/10. doi: 10.1002/prot.24258. PubMed PMID: 23349002.

18. Koehler Leman J, Lyskov S, Bonneau R. Computing structure-based lipid accessibility of membrane proteins with mp_lipid_acc in RosettaMP. BMC bioinformatics. 2017;18(1):115-. doi: 10.1186/s12859-017-1541-z. PubMed PMID: 28219343.
