# Supplementary material for: Membrane contact probability: An essential and predictive character for the structural and functional studies of membrane proteins
Source: PLoS Comput Biol. 2022 Mar 30;18(3):e1009972. doi: 10.1371/journal.pcbi.1009972 (PMC9000120; doi:10.1371/journal.pcbi.1009972)
Supplement: S2 Table — (DOCX) [file pcbi.1009972.s015.docx]

**Table S2: The performance of the MCP predictor using the MCP-Small dataset**

| Evaluation | Training | | Validation | Test |
| --- | --- | --- | --- | --- |
| Overall | | | | |
| MSE | | 0.057 | 0.074 | 0.080 |
| PCC | | 0.705 | 0.656 | 0.635 |
| $\alpha-$helix (H) | | | | |
| MSE | 0.063 | | 0.080 | 0.090 |
| PCC | 0.819 | | 0.766 | 0.728 |
| $\beta-$sheet (E) | | | | |
| MSE | 0.032 | | 0.045 | 0.041 |
| PCC | 0.706 | | 0.598 | 0.575 |
| Coil (C) | | | | |
| MSE | 0.014 | | 0.021 | 0.018 |
| PCC | 0.618 | | 0.446 | 0.429 |
