# Supplementary material for: Membrane contact probability: An essential and predictive character for the structural and functional studies of membrane proteins
Source: PLoS Comput Biol. 2022 Mar 30;18(3):e1009972. doi: 10.1371/journal.pcbi.1009972 (PMC9000120; doi:10.1371/journal.pcbi.1009972)
Supplement: S3 Table — (DOCX) [file pcbi.1009972.s016.docx]

**Table S3: The performance of our MCP predictor for different transmembrane protein classes.**

| Class | Single-pass $\alpha-$helical | Multi-pass $\alpha-$helical | $\beta$-barrel |
| --- | --- | --- | --- |
| MSE (train) | 0.063 | 0.051 | 0.042 |
| PCC (train) | 0.821 | 0.830 | 0.745 |
| MSE (validation) | 0.061 | 0.054 | 0.043 |
| PCC (validation) | 0.816 | 0.818 | 0.700 |
| MSE (test) | 0.060 | 0.055 | 0.043 |
| PCC (test) | 0.800 | 0.818 | 0.728 |
