# Supplementary material for: Membrane contact probability: An essential and predictive character for the structural and functional studies of membrane proteins
Source: PLoS Comput Biol. 2022 Mar 30;18(3):e1009972. doi: 10.1371/journal.pcbi.1009972 (PMC9000120; doi:10.1371/journal.pcbi.1009972)
Supplement: S4 Table — (DOCX) [file pcbi.1009972.s017.docx]

**Table S4: The amount of proteins of different classes in the datasets.**

| Class | Single-pass $\alpha-$helical | Multi-pass $\alpha-$helical | $\beta$-barrel |
| --- | --- | --- | --- |
| MCP-Large | | | |
| Train | 1062 | 2832 | 1106 |
| Validation | 81 | 231 | 88 |
| Test | 103 | 284 | 113 |
| MCP-Small | | | |
| Train | 134 | 367 | 217 |
| Validation | 16 | 44 | 30 |
| Test | 19 | 46 | 25 |
