# Supplementary material for: Membrane contact probability: An essential and predictive character for the structural and functional studies of membrane proteins
Source: PLoS Comput Biol. 2022 Mar 30;18(3):e1009972. doi: 10.1371/journal.pcbi.1009972 (PMC9000120; doi:10.1371/journal.pcbi.1009972)
Supplement: S5 Table — (DOCX) [file pcbi.1009972.s018.docx]

**Table S5: The amino acids predicted with high MCP values in the 102 Pfam soluble protein dataset.**

| Sequence ID | Residue ID (MCP$\geq$0.5) | MCP values |
| --- | --- | --- |
| 1f6bA | M1 | 0.996 |
|  | F3 | 0.947 |
|  | G11 | 0.651 |
|  | F12 | 0.779 |
|  | V15 | 0.854 |
|  | F18 | 0.995 |
| 1mugA | V2 | 0.990 |
|  | Q126 | 0.550 |
| 1rybA | Y66 | 0.550 |
|  | L104 | 0.903 |
| 1gz2A | F98 | 0.927 |
| 1qf9A | M192 | 0.781 |
| 1vfyA | L186 | 0.762 |
| 1vhuA | A14 | 0.506 |
