# Supplementary material for: Membrane contact probability: An essential and predictive character for the structural and functional studies of membrane proteins
Source: PLoS Comput Biol. 2022 Mar 30;18(3):e1009972. doi: 10.1371/journal.pcbi.1009972 (PMC9000120; doi:10.1371/journal.pcbi.1009972)
Supplement: S6 Table — (DOCX) [file pcbi.1009972.s019.docx]

**Table S6: The contact map prediction precision of the additional 495-protein dataset: overall, soluble, and membrane proteins.**

| 495 test proteins in total | | | | | | | | | | | | |
| --- | --- | --- | --- | --- | --- | --- | --- | --- | --- | --- | --- | --- |
| Methods | Short | | | | Medium | | | | Long | | | |
|  | L/10 | L/5 | L/2 | L | L/10 | L/5 | L/2 | L | L/10 | L/5 | L/2 | L |
| ResNet | 0.75 | 0.62 | 0.38 | 0.22 | 0.78 | 0.67 | 0.44 | 0.27 | 0.86 | 0.82 | 0.71 | 0.55 |
| ResNet + MCP | 0.76 | 0.64 | 0.40 | 0.23 | 0.79 | 0.69 | 0.46 | 0.28 | 0.86 | 0.83 | 0.73 | 0.57 |
| 480 soluble proteins | | | | | | | | | | | | |
| Methods | Short | | | | Medium | | | | Long | | | |
|  | L/10 | L/5 | L/2 | L | L/10 | L/5 | L/2 | L | L/10 | L/5 | L/2 | L |
| ResNet | 0.75 | 0.62 | 0.38 | 0.22 | 0.78 | 0.68 | 0.45 | 0.27 | 0.86 | 0.82 | 0.71 | 0.56 |
| ResNet + MCP | 0.76 | 0.64 | 0.40 | 0.23 | 0.80 | 0.69 | 0.46 | 0.28 | 0.86 | 0.83 | 0.73 | 0.57 |
| 15 membrane proteins | | | | | | | | | | | | |
| Methods | Short | | | | Medium | | | | Long | | | |
|  | L/10 | L/5 | L/2 | L | L/10 | L/5 | L/2 | L | L/10 | L/5 | L/2 | L |
| ResNet | 0.59 | 0.47 | 0.27 | 0.15 | 0.64 | 0.53 | 0.30 | 0.17 | 0.82 | 0.79 | 0.67 | 0.50 |
| ResNet + MCP | 0.60 | 0.50 | 0.29 | 0.16 | 0.66 | 0.53 | 0.31 | 0.18 | 0.83 | 0.80 | 0.70 | 0.52 |
