# Supplementary material for: Membrane contact probability: An essential and predictive character for the structural and functional studies of membrane proteins
Source: PLoS Comput Biol. 2022 Mar 30;18(3):e1009972. doi: 10.1371/journal.pcbi.1009972 (PMC9000120; doi:10.1371/journal.pcbi.1009972)
Supplement: S7 Table — (DOCX) [file pcbi.1009972.s020.docx]

**Table S7: The contact map prediction precision for the two representative cases: 5aym and 4e1t.**

| Contact prediction precision for 5aym | | | | | | | | | | | | |
| --- | --- | --- | --- | --- | --- | --- | --- | --- | --- | --- | --- | --- |
| Methods | Short | | | | Medium | | | | Long | | | |
|  | L/10 | L/5 | L/2 | L | L/10 | L/5 | L/2 | L | L/10 | L/5 | L/2 | L |
| CCMpred | 0.16 | 0.09 | 0.05 | 0.04 | 0.07 | 0.05 | 0.04 | 0.03 | 0.34 | 0.18 | 0.10 | 0.08 |
| PSICOV | 0.30 | 0.18 | 0.09 | 0.06 | 0.20 | 0.11 | 0.06 | 0.05 | 0.73 | 0.64 | 0.41 | 0.29 |
| MetaPSICOV | 0.39 | 0.26 | 0.14 | 0.08 | 0.36 | 0.25 | 0.15 | 0.08 | 0.66 | 0.64 | 0.50 | 0.35 |
| MetaPSICOV2 | 0.41 | 0.27 | 0.15 | 0.08 | 0.43 | 0.28 | 0.16 | 0.10 | 0.77 | 0.68 | 0.56 | 0.38 |
| ResNet | 0.48 | 0.32 | 0.16 | 0.09 | 0.55 | 0.35 | 0.20 | 0.11 | 0.86 | 0.76 | 0.58 | 0.48 |
| ResNet + MCP | 0.59 | 0.35 | 0.16 | 0.09 | 0.70 | 0.42 | 0.21 | 0.11 | **0.95** | **0.86** | **0.76** | **0.54** |
| Contact prediction precision for 4e1t | | | | | | | | | | | | |
| Methods | Short | | | | Medium | | | | Long | | | |
|  | L/10 | L/5 | L/2 | L | L/10 | L/5 | L/2 | L | L/10 | L/5 | L/2 | L |
| PSICOV | 0.33 | 0.31 | 0.20 | 0.17 | 0.46 | 0.29 | 0.20 | 0.17 | 0.38 | 0.27 | 0.20 | 0.13 |
| CCMpred | 0.54 | 0.51 | 0.34 | 0.22 | 0.71 | 0.53 | 0.34 | 0.21 | 0.54 | 0.53 | 0.30 | 0.18 |
| MetaPSICOV | 0.79 | 0.82 | 0.59 | 0.38 | 0.83 | 0.82 | 0.68 | 0.49 | 0.58 | 0.43 | 0.36 | 0.23 |
| MetaPSICOV2 | 0.88 | 0.82 | 0.66 | 0.39 | 1.00 | 0.94 | 0.77 | 0.56 | 0.71 | 0.61 | 0.43 | 0.28 |
| ResNet | 1.00 | 0.96 | 0.79 | 0.44 | 1.00 | 0.98 | 0.91 | 0.66 | 1.00 | 0.92 | 0.80 | 0.56 |
| ResNet + MCP | 1.00 | 0.98 | 0.81 | 0.44 | 1.00 | 1.00 | 0.96 | 0.66 | **1.00** | **0.98** | **0.83** | **0.63** |
