# Supplementary material for: Membrane contact probability: An essential and predictive character for the structural and functional studies of membrane proteins
Source: PLoS Comput Biol. 2022 Mar 30;18(3):e1009972. doi: 10.1371/journal.pcbi.1009972 (PMC9000120; doi:10.1371/journal.pcbi.1009972)
Supplement: S8 Table — (DOCX) [file pcbi.1009972.s021.docx]

**Table S8: The RMSD of the top five structure prediction models with respect to the crystal structures for the two representative cases: 5aym and 4e1t.**

| 5aym | | |
| --- | --- | --- |
| Model | RMSD (Å) | |
|  | Without MCP | With MCP |
| 1 | **4.55** | **3.25** |
| 2 | 4.73 | 3.60 |
| 3 | 5.33 | 3.61 |
| 4 | 5.48 | 3.73 |
| 5 | 6.00 | 3.86 |
| 4e1t | | |
| Methods | RMSD (Å) | |
|  | Without MCP | With MCP |
| 1 | **4.12** | **3.41** |
| 2 | 4.32 | 4.05 |
| 3 | 4.43 | 4.09 |
| 4 | 4.56 | 4.10 |
| 5 | 6.33 | 5.25 |
