# Supplementary material for: Membrane contact probability: An essential and predictive character for the structural and functional studies of membrane proteins
Source: PLoS Comput Biol. 2022 Mar 30;18(3):e1009972. doi: 10.1371/journal.pcbi.1009972 (PMC9000120; doi:10.1371/journal.pcbi.1009972)
Supplement: S9 Table — (DOCX) [file pcbi.1009972.s022.docx]

**Table S9: The performance of our MCP predictor for different oligomeric states.**

| State | Monomer | Complex |
| --- | --- | --- |
| MSE (train) | 0.061 | 0.043 |
| PCC (train) | 0.830 | 0.757 |
| MSE (validation) | 0.064 | 0.046 |
| PCC (validation) | 0.820 | 0.746 |
| MSE (test) | 0.069 | 0.042 |
| PCC (test) | 0.799 | 0.761 |
