# Supplementary material for: Membrane contact probability: An essential and predictive character for the structural and functional studies of membrane proteins
Source: PLoS Comput Biol. 2022 Mar 30;18(3):e1009972. doi: 10.1371/journal.pcbi.1009972 (PMC9000120; doi:10.1371/journal.pcbi.1009972)
Supplement: S10 Table — (DOCX) [file pcbi.1009972.s023.docx]

**Table S10: The performance of our MCP predictor in the 10-fold cross-validation using the MCP-Large dataset.**

| Evaluation | Training | Test |
| --- | --- | --- |
| Overall | | |
| MSE | 0.051$\pm$0.004 | 0.053$\pm$0.005 |
| PCC | 0.786$\pm$0.018 | 0.774$\pm$0.017 |
| $\alpha-$helix (H) | | |
| MSE | 0.058$\pm$0.005 | 0.061$\pm$0.006 |
| PCC | 0.834$\pm$0.017 | 0.823$\pm$0.019 |
| $\beta-$sheet (E) | | |
| MSE | 0.028$\pm$0.002 | 0.029$\pm$0.003 |
| PCC | 0.717$\pm$0.028 | 0.696$\pm$0.033 |
| Coil (C) | | |
| MSE | 0.013$\pm$0.001 | 0.014$\pm$0.002 |
| PCC | 0.585$\pm$0.033 | 0.564$\pm$0.033 |
