# Supplementary material for: Membrane contact probability: An essential and predictive character for the structural and functional studies of membrane proteins
Source: PLoS Comput Biol. 2022 Mar 30;18(3):e1009972. doi: 10.1371/journal.pcbi.1009972 (PMC9000120; doi:10.1371/journal.pcbi.1009972)
Supplement: S11 Table — (DOCX) [file pcbi.1009972.s024.docx]

**Table S11: The performance of our MCP predictor in the 10-fold cross-validation using the MCP-Small dataset.**

| Evaluation | Training | Test | |
| --- | --- | --- | --- |
| Overall | | |  |
| MSE | 0.055$\pm$0.005 | 0.077$\pm$0.007 | |
| PCC | 0.703$\pm$0.020 | 0.634$\pm$0.050 | |
| $\alpha-$helix (H) | | |  |
| MSE | 0.064$\pm$0.006 | 0.088$\pm$0.007 | |
| PCC | 0.819$\pm$0.019 | 0.744$\pm$0.017 | |
| $\beta-$sheet (E) | | |  |
| MSE | 0.032$\pm$0.002 | 0.045$\pm$0.004 | |
| PCC | 0.711$\pm$0.026 | 0.567$\pm$0.019 | |
| Coil (C) | | |  |
| MSE | 0.015$\pm$0.001 | 0.019$\pm$0.003 | |
| PCC | 0.607$\pm$0.030 | 0.440$\pm$0.038 | |
