# Supplementary material for: Membrane contact probability: An essential and predictive character for the structural and functional studies of membrane proteins
Source: PLoS Comput Biol. 2022 Mar 30;18(3):e1009972. doi: 10.1371/journal.pcbi.1009972 (PMC9000120; doi:10.1371/journal.pcbi.1009972)
Supplement: S12 Table — (DOCX) [file pcbi.1009972.s025.docx]

**Table S12: The basic statistics of the datasets according to the database SCOPe [1, 2].**

| Dataset | All alpha | All beta | Alpha/beta | Alpha+beta | Others | Sequence identity |
| --- | --- | --- | --- | --- | --- | --- |
| 10054 proteins | 641 | 604 | 676 | 934 | 7199 | 25% |
| 327 test proteins | 21 | 26 | 36 | 25 | 219 | 25% |
| 495-protein dataset | 22 | 32 | 21 | 28 | 392 | 25% |

**Reference**

1. Fox NK, Brenner SE, Chandonia J-M. SCOPe: Structural Classification of Proteins--extended, integrating SCOP and ASTRAL data and classification of new structures. Nucleic acids research. 2014;42(Database issue):D304-D9. Epub 2013/12/03. doi: 10.1093/nar/gkt1240. PubMed PMID: 24304899.

2. Chandonia J-M, Fox NK, Brenner SE. SCOPe: classification of large macromolecular structures in the structural classification of proteins-extended database. Nucleic acids research. 2019;47(D1):D475-D81. doi: 10.1093/nar/gky1134. PubMed PMID: 30500919.
