# Supplementary material for: Membrane contact probability: An essential and predictive character for the structural and functional studies of membrane proteins
Source: PLoS Comput Biol. 2022 Mar 30;18(3):e1009972. doi: 10.1371/journal.pcbi.1009972 (PMC9000120; doi:10.1371/journal.pcbi.1009972)
Supplement: S13 Table — (DOCX) [file pcbi.1009972.s026.docx]

**Table S13: The performance (precision of medium- and long-range contact) of our contact map predictor in the 10-fold cross-validation.**

| Methods | Medium | | | | Long | | | |
| --- | --- | --- | --- | --- | --- | --- | --- | --- |
|  | L/10 | L/5 | L/2 | L | L/10 | L/5 | L/2 | L |
| ResNet | 0.795  $\pm$0.007 | 0.697  $\pm$0.010 | 0.476  $\pm$0.011 | 0.296  $\pm$0.006 | 0.852  $\pm$0.007 | 0.805  $\pm$0.007 | 0.707  $\pm$0.010 | 0.550  $\pm$0.011 |
| ResNet + MCP | 0.832  $\pm$0.007 | 0.730  $\pm$0.008 | 0.496  $\pm$0.008 | 0.306  $\pm$0.005 | **0.895**  $\pm$0.007 | **0.860**  $\pm$0.007 | **0.758**  $\pm$0.006 | **0.608**  $\pm$0.006 |
