# Supplementary material for: Membrane contact probability: An essential and predictive character for the structural and functional studies of membrane proteins
Source: PLoS Comput Biol. 2022 Mar 30;18(3):e1009972. doi: 10.1371/journal.pcbi.1009972 (PMC9000120; doi:10.1371/journal.pcbi.1009972)
Supplement: S14 Table — (DOCX) [file pcbi.1009972.s027.docx]

**Table S14: The normalized confusion matrix of the contact map prediction (cutoff=0.5).**

| Dataset | Labels | | Observation | |
| --- | --- | --- | --- | --- |
|  |  |  | 0 | 1 |
| 327 test proteins | Prediction  (ResNet) | 0 | 0.993 | 0.662 |
|  |  | 1 | 0.007 | 0.338 |
|  | Prediction  (ResNet+MCP) | 0 | 0.995 | **0.595** |
|  |  | 1 | 0.005 | **0.405** |
| 495-protein dataset | Prediction  (ResNet) | 0 | 0.993 | 0.649 |
|  |  | 1 | 0.007 | 0.351 |
|  | Prediction  (ResNet+MCP) | 0 | 0.993 | **0.633** |
|  |  | 1 | 0.007 | **0.367** |
