# Supplementary material for: Membrane contact probability: An essential and predictive character for the structural and functional studies of membrane proteins
Source: PLoS Comput Biol. 2022 Mar 30;18(3):e1009972. doi: 10.1371/journal.pcbi.1009972 (PMC9000120; doi:10.1371/journal.pcbi.1009972)
Supplement: S15 Table — (DOCX) [file pcbi.1009972.s028.docx]

**Table S15: The accuracy (cutoff=0.5) and AUC of the contact map prediction.**

| Dataset/ Evaluation | Accuracy | | AUC | |
| --- | --- | --- | --- | --- |
| Test set | ResNet | ResNet+MCP | ResNet | ResNet+MCP |
| 327 test proteins | 0.961 | **0.968** | 0.932 | **0.954** |
| 495-protein dataset | 0.966 | **0.969** | 0.933 | **0.945** |
